# Maintenance Therapy in AML

**DOI:** 10.3389/fonc.2020.619085

**Published:** 2021-02-02

**Authors:** Patrick K. Reville, Tapan M. Kadia

**Affiliations:** Department of Leukemia, University of Texas MD Anderson Cancer Center, Houston, TX, United States

**Keywords:** acute myeloid leukemia, maintenance therapy, cancer, targeted therapy, chemotherapy, immunotherapy

## Abstract

Recent advances in therapeutics coupled with steady improvements in supportive care for patients with acute myeloid leukemia (AML) have led to improved outcomes. Despite these advances, even in patients that achieve a complete remission with initial therapy high rates of relapse remain a clinical dilemma. For decades, investigators have attempted strategies of maintenance therapy to prolong both remission duration and overall survival in patients with AML. These approaches have included cytotoxic chemotherapy, immunotherapy, hypomethylating agents, and targeted small molecule therapy. Overall, the evidence in favor of maintenance therapy is limited. Recent strategies, especially with hypomethylating agents have begun to show promise as maintenance therapy in improving clinical outcomes. Ongoing and future studies will continue to elucidate the true role for maintenance therapy options in patients with AML. In this review we summarize prior and ongoing maintenance therapy approaches in AML and highlight some of the most promising strategies.

## Introduction

Advances in therapeutics and supportive care for acute myeloid leukemia (AML) have led to steady improvements in the outcomes for patients with AML. For example, dose intensification and novel drug combinations during induction therapy have led to higher response rates and improved survival in patients with newly diagnosed AML (1–6). Consolidation therapy helps to eradicate residual leukemia and reduces the risk of disease relapse (2, 7, 8). Despite these successes, relapse remains a major concern with relapse risk greater than 50% for all adults with high risk AML (9–11). There is a critical need for therapeutic strategies that decrease this relapse risk and improve the survival of patients with AML.

Patients with high risk AML that are ineligible for allogeneic hematopoietic stem cell transplantation (alloSCT) continue to have poor outcomes and low likelihood of cure (12). The most effective post remission therapy in AML continues to be alloSCT, but is not available to all patients with high-risk disease because high rates of complications limit broad applicability to patients with multiple comorbidities and some patients lack suitable donors (8, 13–15). A major reason for the success of alloSCT in maintenance of remission and cure of AML is through the generation of allo-reactive T cells and graft versus leukemia (GVL) effect (16–18). The current standard of care for most patients with AML achieving a CR with induction and consolidation is observation without maintenance therapy, with the exception of acute promyelocytic leukemia where maintenance arsenic trioxide and retinoic acid have shown clear benefit (19, 20). However with the recent completion of the QUAZAR AML-001 clinical study and FDA approval of CC-486 (oral azacitidine) this paradigm maybe set to change (21).

Given the high rate of relapse, there is rationale and need for post remission maintenance therapy to mitigate this risk. Maintenance remains a standard of care for patients with acute lymphoblastic leukemia (22), yet even in ALL maintenance therapy with 6-mercaptopurine, methotrexate, vincristine, and prednisone (POMP) has never been formally tested in randomized studies. Despite this, studies consistently show inferior outcomes in ALL without maintenance therapy (23–25). Clinical trials evaluating maintenance cytotoxic chemotherapy in AML in the past have consistently failed to show a benefit in overall survival while providing occasionally seen benefit in relapse-free survival (26–29).

The goal of maintenance therapy should be to improve overall survival. Improvements in disease-free, relapse-free, or event-free survival are not enough to justify the added exposure to and toxicity from anti-leukemia therapy unless they translate to gains in overall survival. This is especially true when effective salvage treatment options exist. Active therapy where active disease exists is likely to delay relapse; but if observation followed by salvage therapy leads to similar overall survival then we are likely increasing risk of therapy side effects without meaningfully affecting the natural history of a patient’s AML. Relatively few clinical trials have met this bar. With the availability of newer methods to measure minimal residual disease (MRD) after achieving a complete morphologic response, it seems intuitive that this residual disease that persists after induction and consolidation is the source of most relapses. It follows, then, that another quantifiable goal of post remission maintenance therapy is to eradicate MRD. In this review we summarize the clinical trial data on maintenance therapy in adults with AML as well as highlight avenues of promising research.

## Maintenance Cytotoxic Chemotherapy

Similar to ALL, the first post-consolidation maintenance approaches in AML involved the continued use of similar cytotoxic chemotherapy to prolong remission. There have been five main randomized studies that investigated maintenance chemotherapy compared with observation in patients with AML that is in a complete remission (CR) (26, 28, 30–32). The Swiss Group for Clinical Cancer Research study randomized 74 patients with AML in remission following induction and consolidation treatment to maintenance therapy with every 8 weeks for 2 years or observation (26). The maintenance regimen consisted of alternating cytarabine (ara-C) and 6-thioguanine with ara-C and prednisone, which showed no difference in relapse or survival compared with observation (26). The German AML Cooperative Group randomized 145 patients with AML after 6-thioguanine, ara-C, and daunorubicin (TAD) induction and consolidation to a maintenance strategy of monthly alternating courses of cytarabine-daunorubicin, cytarabine–6-thioguanine, cytarabine-cyclophosphamide for 3 years (30). This strategy improved relapse-free survival (RFS) but the report did not mention overall survival (OS) (30). In a small study from the SW Leukemia Group, 32 patients were randomly assigned to maintenance therapy with monthly thioguanine and etoposide alternating with lomustine for a total of 6 courses which did not affect remission duration (31). In a study from EORTC and HOVON, 147 patients with AML in remission were randomized to maintenance low dose ara-C (LDAC) or observation (28). Maintenance LDAC improved disease-free survival (DFS) but did not improve overall survival (28). In a Group LAME study the addition of maintenance therapy with continuous oral mercaptopurine and monthly pulses of LDAC for patients in CR failed to improve 5-year DFS and resulted in inferior overall survival attributed to lower rate of response to salvage therapy (32).

Several other studies have incorporated maintenance chemotherapy strategies into their design however these have not included observation-only control arms (33–45). Given the unclear benefit of adding maintenance cytotoxic chemotherapy to patients with AML in a CR, the lack of an observation-only control arm makes these trials difficult to interpret in this setting. Based on the available data there is no consistent role for maintenance cytotoxic chemotherapy in adult AML patients in a CR given the lack of overall survival benefit and inconsistent benefit of RFS/DFS. Many of these studies are plagued by a high level of both treatment and disease heterogeneity and small sample sizes, which make any potential benefit difficult to interpret in the setting of modern AML diagnosis and treatment.

## Maintenance Hypomethylating Agents

Recent studies with maintenance hypomethylating agents (HMA) have shown some promise in patients with AML in CR that are not eligible for alloSCT. Three major randomized studies have compared strategies using azacitidine maintenance with observation (21, 46, 47). In the UK NCRI AML16 trial participants achieving a CR were randomized to azacitidine 75 mg/m^2^ per day on days 1 to 5 for nine 6-week courses compared with observation (46). The addition of maintenance azacitidine did not result in improvement in OS; yet an unplanned subset analysis showed that for patients with no measurable residual disease (MRD) there appeared to be a survival benefit with 5-year OS of 40% in the azacitidine arm and 13% in the observation arm; this was not observed in patients with measurable MRD (46). The HOVON97 phase 3 randomized trial enrolled patients > 60 years of age with AML or MDS in CR/CRi and treated them with azacitidine 50 mg/m2 subcutaneously, days 1–5, every 4 weeks or placebo until disease progression (47). Median disease free survival in the maintenance azacitidine arm was 15.9 months which was improved from the placebo arm (HR: 0.62; 95% CI: 0.41–0.95), however this did not confer an overall survival advantage of maintenance azacitidine (HR: 0.91; 95% CI: 0.58–1.44) (47). In an exploratory multivariate analysis of this study, the DFS benefit of maintenance azacitidine appeared to be limited to patients with a platelet count of at least 100 × 10^9^/L and those in a CR at inclusion, where the DFS benefit was not seen for those with platelet count less than 100 × 10^9^/L and in CRi at inclusion (47).

One randomized study has compared decitabine maintenance with observation, the ECOG-ACRIN E2906 study was a randomized phase II trial which enrolled AML patients ≥ 60 years of age in CR/CRi after induction and consolidation therapy and randomized them to decitabine 20 mg/m2 on days 1–3 every 28 days for 1 year or observation (48). The study was closed with only 70% of target accrual which limited power; here they showed median disease free survival of 15.3 months in decitabine arm (HR: 0.77, 95% CI: 0.50–1.19) and median overall survival of 25.8 months (HR: 0.69, 95% CI: 0.43–1.09) both favoring the decitabine arm but neither reaching statistical significance (48). A randomized study of decitabine versus conventional care for maintenance therapy (which included LDAC, prolonged intensive chemotherapy, or observation) in patients with acute myeloid leukemia in complete remission was completed which ultimately failed to show a benefit to maintenance decitabine (49).

QUAZAR AML-001 study demonstrated the superiority of CC-486, an oral formulation of azacitidine, over placebo as a maintenance strategy in AML patients >55 years of age in first remission (21). This study enrolled patients who were aged 55 years or older, had AML, and were within 4 months of achieving first complete remission (CR) or complete remission with incomplete blood count recovery (CRi) with intensive induction chemotherapy and were not candidates for HSCT. In this study, CC-486 was given at 300 mg daily on days 1–14 of a 28-day cycle and continued until disease progression, unacceptable toxicity, or alloSCT. CC-486 improved both the relapse free and overall survival compared with placebo with median relapse free survival of 10.2 months (HR: 0.66; 95% CI: 0.53–0.81) and median overall survival of 24.7 months (HR: 0.65; 95% CI: 0.52–0.81) in the CC-486 arm (21). The subgroup analysis for overall survival showed that the point estimates for all subgroups favored the CC-486 arm. With that being said there are a few subgroups that appeared to have preferential benefit from maintenance CC-486: those that were ≥65 years old, those in a CR, those that did not receive consolidation therapy, and those that were MRD positive at study inclusion. The ideal patient for this type of maintenance approach might be an older patient, not a candidate for alloSCT, that was unable to receive any or the intended amount of consolidation therapy that is in an MRD positive state.

Overall, in patients with AML in CR that are ineligible for alloSCT, maintenance HMA approaches look promising. Now for the first time, the investigational agent CC-486 has shown an overall survival benefit using an oral azacitidine regimen; however we await the full publication before declaring this as the de facto standard of care despite recent FDA approval (21, 50). The other maintenance HMA approaches have generally shown promise in delaying relapse, yet they have consistently failed to improve overall survival. This coupled with their parenteral delivery and need for frequent clinic visits likely makes them less desirable for maintenance therapy in the majority of patients.

## Maintenance Immunotherapies

Probably the most extensively studied approach to maintenance therapy in patients with AML has been with immunotherapy. In some ways, alloSCT can be considered a type of maintenance therapy in that grafted allogeneic T cells continuously surveille and maintain remission in responders through GVL effect. AlloSCT serves as a proof of concept that AML is an immune responsive malignancy and that harnessing the immune system has the potential to cure AML. However, early approaches with BCG, interleukin-2, and interferon alpha have failed to show consistent benefits of maintenance immunotherapy in AML.

There have been four RCTs on the value of adding BCG vaccination as maintenance therapy in patients with AML. Only one study, which used a combination vaccination approach with BCG and allogeneic AML cells, showed an improvement in both remission duration and OS; however that study only recruited 41 patients (51). The other three RCTs of BCG vaccination approaches failed to show benefits of this approach as maintenance therapy in patients with AML (52–54).

Cytokine therapy with interleukin-2 (IL-2) and interferon alpha (IFNα) have also been studied in a number of RCTs. Seven major RCTs have evaluated the role of IL-2 as maintenance therapy in patients with AML compared with observation (55–61). A patient level meta-analysis of six studies was performed in 2011 (62) and updated in 2015 (63), showed no benefit in terms of DFS or OS with this approach, as did the seventh trial published in 2018. IL-2 plays a crucial role in cytotoxic T and NK cell growth and survival which serves as rationale for its use in stimulating anti-leukemic immune responses. However it is also crucial for regulatory T cell survival, which may help explain the lack of clinical benefits seen, especially with low-dose IL-2 therapy (64–66). IL-2 combined with histamine dihydrochloride (HDC) is approved by the European Medicines Agency for remission maintenance in adult patients with AML in CR1 (67). This is based on the results of a Swedish study of 320 patients with AML in CR randomized to IL-2/HDC or observation which improved leukemia-free survival (LFS), especially in those in CR1 where 3-year LFS was 40% in the IL-2/HDC arm and 26% in the observation arm (68). The benefits in LFS did not translate to improvements in OS (68). Despite the lack of OS benefit, this therapy appeared to be very well tolerated with 92% of non-relapsed patients completing all intended cycles (10) of therapy (68). A German study (NCT01770158) that was set to investigate MRD dynamics with Il-2/HDC therapy in adult AML patients in CR but with MRD, however has been closed due to inability to accrue (NCT01770158). A similarly designed Swedish study (NCT01347996) to evaluate MRD in patients receiving IL-2/HDC has completed but has not yet reported results (NCT01347996).

The other cytokine that has been studied as a maintenance strategy for AML is IFNα, which may have both direct activity against AML blasts as well as indirect action through immune stimulation (69). Despite the biologic rationale, RCTs of IFNα have failed to show a benefit of IFNα as a maintenance strategy in patients with AML in CR (27, 70). A Finnish study randomized patients with AML in CR to either observation, IFNα maintenance, or thioguanine and ara-C maintenance and found no difference in these arms in terms of OS (27). The UK MRC AML11 trial included a maintenance phase (12 months with low-doses IFNα) for 362 older AML patients in CR1, which added no improvement in DFS or OS (70).

Over the last decade the development of immune checkpoint inhibitors that block immune inhibitory molecules, PD-1, PD-L1, and CTLA-4, can induce anti-cancer immune responses and have led to dramatic responses in a number of solid tumors such as melanoma and non-small cell lung cancer (71–73). Following the alloSCT example of T cells mediating graft versus leukemia effect, the use of checkpoint blockade to induce autologous, anti-leukemic T cell responses has rationale. We recently concluded a single arm study of nivolumab maintenance in AML patients in CR, at high risk for relapse but ineligible for alloSCT (74). Fifteen patients were treated with the 12- and 24-month estimated overall survival of 60% and 53%, respectively (74). Two patients of nine with detectable MRD at study entry achieved MRD negativity while on therapy; however most patients with detectable MRD at enrollment had progressive disease with eventual disease recurrence (74). A larger, randomized phase 2 study (NCT02275533) of nivolumab for eradication of MRD in high-risk AML in remission is ongoing and should further clarify this strategy.

The combination of HMA and nivolumab may have additive effects in the maintenance setting.

There is rationale for the combination of HMAs and immune checkpoint blockade, with demethylation of PD1 promoter associated with worse response in MDS and AML and increased expression of immune checkpoint molecules in patients with MDS treated with HMA (75, 76). The combination of nivolumab and azacitidine in the relapsed/refractory AML setting was recently reported and showed an overall response rate of 33%, with the overall response rate in the HMA-naive patients of 58% (77).

Lenalidomide is an orally bioavailable cereblon modulator, approved for the treatment of myelodysplastic syndrome and multiple myeloma. Lenalidomide, which as shown activity in AML, has also been shown to enhance natural killer (NK) cell cytotoxicity, cytokine secretion, and immune synapse formation that may favor anti-leukemia immunity. A recent single-arm phase II study of lenalidomide maintenance in patients with high-risk AML in CR1 or CR2, ineligible for SCT, reported interesting results (78). Among 28 patients, with a median follow-up of 22.3 months, the median CR duration was 18.7 months and the 2-year OS was 63%, surpassing historical controls (78). The benefit was most pronounced in patients with non-secondary AML and those who had undetectable MRD (78). While promising, a larger, randomized study is needed to confirm its benefit.

Despite intense focus of research over decades and strong preclinical and clinical rationale, maintenance immunotherapies have been generally disappointing. These studies have mostly suffered from heterogeneous patient populations and small sample sizes. None have shown a clear benefit in overall survival and only IL-2/HDC has a major drug approval in maintenance therapy. Ongoing efforts with checkpoint inhibitors potentially combined with HMAs or other agents such as targeted bispecific antibodies may show benefits in the future.

## Maintenance Targeted Therapies

FLT3 inhibitors have been incorporated into studies that included maintenance FLT3-inhibitors in their design, in treating patients with FLT3 mutated AML. In general, it has been difficult within these studies, to ascertain the relative benefit of the maintenance phase of FLT3 inhibitors compared with the benefit in the induction and consolidation phases. With that caveat, the RATIFY trial assessed midostaurin at a dose of 50 mg twice daily added to induction and consolidation therapy and then followed by 12 months of maintenance midostaurin compared to standard therapy that did not include maintenance therapy (2). Addition of midostaurin to standard therapy resulted in significant improvements in both OS (74.7 months vs 26.0 months) and event-free survival (EFS)(8.0 months vs 3.0 months) which were also significant when censoring for alloSCT (2). In an unplanned post-hoc analysis of the RATIFY trial, the relative benefit of maintenance midostaurin on DFS could not be definitively ascertained. Here the authors looked at patients that went on to the maintenance phase of treatment, the point estimate for DFS hazard ratio was 0.83 when comparing midostaurin maintenance to placebo but with a wide confidence interval consistent with both large benefit as well as potential harm (95%CI, 0.48–1.43) (79). Additionally, a recently completed single arm German-Austrian AML Study Group trial investigated midostaurin added to chemotherapy and continued as a single-agent maintenance therapy in AML with FLT3-ITD (80). Here, 34% of the trial population (97 patients) went on to receive maintenance midostaurin either after alloSCT or consolidation and in a propensity matched analysis of the overall trial population compared with historical controls midostaurin improved EFS (80). While still unclear how much benefit the maintenance phase is adding, if treating patients per the RATIFY protocol, continuation of midostaurin maintenance post consolidation is recommended. Similarly, the SORAML trial randomized patients with FLT3 mutated AML to standard therapy with or without sorafenib (at a dose of 400 mg twice daily); for patients in CR1 the protocol added maintenance sorafenib or placebo (81). Patients that received sorafenib had improved EFS of 21 months vs 9.5 months but no improvement in OS (81). A similar trial assessed sorafenib added to standard therapy in older patients with FLT3 altered AML, however in this cohort sorafenib did not appear to improve EFS or OS (82). Gilteritinib which has shown improved outcomes in relapsed/refractory FLT3 mutated AML (83) is also being studied in an ongoing phase 2 study (NCT02927262) where patients are randomized to receive gilteritinib or placebo for a 2-year period after completion of induction/consolidation chemotherapy (83).

Multiple other targeted therapies are being tested in patients with AML with specific molecular alterations, some of which include a maintenance targeted-therapy phase. Many of these studies are single arm studies and when compared with placebo they usually include the targeted therapy in induction, consolidation, and maintenance phases making the specific contribution of the maintenance phase less clear. For example an ongoing phase 1 study (NCT02632708) of patients with IDH1 or IDH2 mutated AML receive ivosidenib (for IDH1 mutated) or enasidenib (for IDH2 mutated) combined with standard therapy for newly diagnosed AML and can continue ivosidenib or enasidenib maintenance until relapse, unacceptable toxicity, or alloSCT. Venetoclax, a bcl-2 inhibitor which has demonstrated improved outcomes in combination with HMAs for older and unfit patients with newly diagnosed AML is also being tested in a phase 2 study (NCT03466294) where patients are treated with azacitidine and venetoclax until MRD negativity is achieved and followed by venetoclax maintenance. A second study is studying a lower dose of azacitidine and venetoclax as post-consolidation maintenance therapy regardless of the type of induction therapy received (NCT04062266). Dasatinib added to intensive induction and consolidation chemotherapy and administered as single agent for 1-year maintenance for first-line patients with core binding factor showed activity in phase Ib/IIa testing and is currently being evaluated in a larger phase 3 study (NCT02013648) (84).

Targeted therapy approaches hold tremendous promise in treating patients with AML. Incorporation of maintenance continuation phases with the oral targeted therapy until relapse, unacceptable toxicity, or alloSCT is an attractive strategy. For example, this was the approval path for midostaurin in patients with FLT3-mutated AML. However, the relative contribution of the maintenance phase is unclear and will be similarly unclear if the same strategy is used for other targeted therapies. Ideally, building in a second randomization into front-line trials studying maintenance vs. observation-only could help answer these questions definitely. This approach becomes a bit more challenging after the recent approval of CC-486 as AML maintenance given the question of an appropriate control arm.

## Post Allogenic Stem Cell Transplant Maintenance Therapies

AML relapse after alloSCT remains a major concern, with approximately 40% of AML patients relapsing post alloSCT and face a dismal prognosis (85–87). We consider maintenance therapy in this setting to mean treatment of patients with negative MRD with the goal of maintaining remission to allow time for or to cooperate with the graft versus leukemia effect to eradicate residual leukemic cells.

The best studied approaches to date are targeted therapies with FLT3 inhibitors. The only published RCT in this setting is the SORMAIN trial which randomized 83 patients with FLT3-ITD–positive AML in CR after alloSCT to 24 months of sorafenib (n = 43) or placebo (n = 40) (88). This study showed an improvement in RFS in favor of the sorafenib group with a HR 0.39 (95% CI, 0.18–0.85) (88). With incomplete OS follow-up there was not a statistically significant benefit in OS, but 2-year landmark survival analysis was improved in the sorafenib arm with 91% of patients in the sorafenib arm alive compared with 66% of patients alive in the placebo arm corresponding to a HR for death of 0.241 (95% CI, 0.08–0.74) (88). A similar phase 2 RCT, RADIUS (NCT01883362) using midostaurin as post alloSCT maintenance has completed but not yet reported. A much larger (n=356) phase 3 RCT of gilteritinib (NCT02997202) as a maintenance strategy in the post alloSCT setting is underway. In the ADMIRAL study, patients undergoing alloSCT that continued gilteritinib appeared to have improved survival (83). Other targeted therapy maintenance strategies are being tested such as the use of enasidenib maintenance after alloSCT in patients with IDH2 mutated myeloid neoplasms (NCT03515512).

Maintenance HMA approaches post-SCT have also been studied. A recently completed study of azacitidine maintenance in high-risk AML and MDS patients post-transplant failed to show a benefit in RFS or OS (89). In an early phase clinical trial, 30 patients with AML received CC-486 (oral azacitidine) maintenance therapy after alloSCT with a 1 year relapse rate of 21% (90). A phase 3 RCT, the AMADEUS study (NCT04173533) of this approach is currently underway. Several other small, single arm studies have tested maintenance HMA in the post alloSCT setting with varying success (91–95). A study combining venetoclax with azacitidine as maintenance therapy post alloSCT is ongoing (NCT04128501).

Immunotherapy approaches have also been studied. Cellular therapy options that have been tested include prophylactic donor lymphocyte infusion (DLI) and NK cells. Prophylactic DLI tested in 62 patients resulted in a 5-year PFS of 65% and OS of 80% (96). Prophylactic NK cell infusion was tested but did not appear to improve relapse rates compared with historical controls (97). A randomized phase 2 vaccination strategy with GVAX (an autologous cancer vaccine with GM-CSF) is currently underway (NCT01773395). Lenalidomide, an immunomodulatory molecule with particular activity in myeloid malignancies with loss of chromosome 5q (del5q), has been tested in a phase 2 LENAMAINT trial, in which 10 patients with del5q AML or MDS were treated with lenalidomide after alloSCT (98). This trial was stopped prematurely because of a signal that lenalidomide increased GVHD with 6/10 patients developing grades 3–4 GVHD within the first two cycles of lenalidomide (98).

## Conclusion

Despite decades of intense study, optimal maintenance therapy in AML has remained elusive. Improvements in overall survival and quality of life (QoL) remain the gold standard bar to achieve for maintenance approaches in patients with AML. Very few maintenance studies have incorporated QoL assessment and outcomes into their design. While we suspect that QoL will be generally diminished when comparing maintenance therapy with observation, this remains to be seen and there may be underappreciated QoL benefits in eliminating residual leukemia, even if not detectable. For now, the goal of maintenance therapy should remain to improve overall survival, but the time to read out survival improvement among patients in remission can slow progress in this arena. Surrogate measures such as eradication of MRD and improvements in relapse-free survival can be early thresholds of success that highlight promising approaches. While randomized trials are the benchmark for clinical benefit, carefully conducted pilot phase II trials with surrogate markers and historical comparisons can also be useful to quickly identify new paradigms. **Table 1** highlights and summarizes the studies which have tested maintenance strategies in placebo controlled randomized studies and their effect on disease, relapse, or leukemia free survival and overall survival.

**Table 1:** Summary of placebo controlled, randomized studies of maintenance therapy in AML.

Trial	Reference Number	Number of patients entering maintenance randomization	Age (Range)	Maintenance treatment regimen	Follow up	Disease/Relapse/Leukemia Free Survival (DFS/RFS/LFS)	Overall Survival (OS)
**HMA**							
UK NCRI AML16	(46)	530	53–84	Azacitidine vs placebo	50.4 months	Not reported	No significant difference in OS
HOVON97	(47)	116	60–81	Azacitidine vs placebo	41.4 months	Median DFS 15.9 months in azacitidine arm vs 10.3 months in placebo arm	No significant difference in OS
QUAZAR AML-001	(21)	472	55–86	CC-486 (oral azacitidine) vs placebo	41.2 months	Median RFS 10.2 months in CC-486 arm vs 4.8 months in placebo arm (p = 0.0001)	Median OS 24.7 months in CC-486 arm vs 14.8 months in placebo arm (p = 0.0009)
ECOG-ACRIN E2906	(48)	120	60–85	Decitabine vs placebo	49.8 months	No significant difference in DFS	No significant difference in OS
**Cytotoxic Chemotherapy**							
Swiss Group for Clinical Cancer Research	(26)	74	7–65	Ara-C-thioguanine alternating with Ara-C-predinsone-vincristine vs placebo	44 months	No significant difference in DFS	No significant difference in OS
German AML Cooperative Group	(30)	145	15–78	Ara-C-daunorubicin alternating with Ara-C–thioguanine and Ara-C-cyclophosphamide vs placebo	2.5 years	Median RFS 13 months in maintenance arm vs 8 months in the nonmaintenance arm (p = 0.003)	Not Reported
SW Leukemia Group	(31)	32	18–74	Thioguanine and etoposide alternating with CCNU vs placebo	Not Reported	No significant difference in DFS	Not Reported
EORTC HOVON	(28)	147	60–88	Low dose Ara-C vs placebo	6 years	Median DFS 51 weeks in Ara-C arm vs 29 weeks in no-Ara-C arm (p = 0.006)	No significant difference in OS
Finnish Leukemia Group	(27)	108	16–59	Ara-C-thioguanine vs human leukocyte IFN vs placebo	82 months	No significant difference in DFS between any of the three arms	No significant difference in OS between any of the three arms
Group LAME	(32)	70	<20 (range not reported)	Oral mercaptopurine and monthly pulses of subcutaneous cytarabine vs placebo	Not Reported	No significant difference in DFS	OS inferior in maintenance arm (p=0.04)
**Immunotherapy**							
Manchester Royal Infirmary	(51)	41	Adult patients (median or range not reported)	BCG and irradiated allogeneic myeloblasts vs placebo	Not Reported	Median RFS 35.14 weeks in maintenance arm vs 19.71 weeks in no maintenance arm (p = 0.039)	Median OS 96.14 weeks in maintenance arm vs 53 weeks in no maintenance arm 9p = 0.045)
Finnish Leukemia Group	(27)	108	16–59	Ara-C-thioguanine vs human leukocyte IFN vs placebo	82 months	No difference in DFS between any of the three arms	No significant difference in OS between any of the three arms
UK MRC AML11	(69)	362	44–75	IFN-α vs placebo	Not Reported	No significant difference in DFS	No significant difference in OS
CALGB 9720	(57)	163	60–83	IL-2 vs placebo	Not Reported	No significant difference in DFS	No significant difference in OS
AML-12	(60)	550	15–60	IL-2 vs placebo	3.6 years	No significant difference in DFS	No significant difference in OS
ALFA-9801	(59)	161	50–70	IL-2 vs placebo	49 months	No significant difference in DFS	No significant difference in OS
ELAM02	(61)	154	<18 (range not reported)	IL-2 vs placebo	49 months	No significant difference in DFS	No significant difference in OS
Brune et al.	(68)	160	18–84	Histamine dihydrochloride plus IL-2 vs placebo	47 months	36 month LFS of 34% in HDC/IL-2 arm vs 24% in placebo arm (p = 0.01)	No significant difference in OS

The biggest advance in AML maintenance currently has been the approval of CC-486, oral azacitidine, demonstrating improvement in both RFS and OS for patients in CR1 that are ineligible for alloSCT. While this is encouraging, the data needs to be examined further and the experience needs to be built upon. When discussing maintenance therapy in AML going forward, it will be important to clarify the role of post induction consolidation. In the QUAZAR-AML-001 study the majority of patients enrolled received either 0 or 1 cycle of consolidation therapy before starting CC-486 (21). Similarly in the UK NCRI AML16 trial, OS was improved incrementally by consolidation or maintenance but not by both (46). With agents like CC-486 or HMAs, are we simply providing a prolonged lower-intensity consolidation (instead of repeated cycles of HiDAC), or is there a benefit to long term maintenance after optimal consolidation therapy? A post-hoc analysis of the QUAZAR-AML-001 study has attempted to answer this question and suggests a benefit of CC-486 regardless of amount of consolidation received, however the overall survival benefit was not statistically longer in the patients receiving one of ≥2 consolidation cycles (99). Targeted therapies have great promise in personalizing AML therapy and improving outcomes. Incorporation of individualized targeted therapies systematically as maintenance post-consolidation is the next frontier. In the post alloSCT setting, the use of maintenance sorafenib appears to improve both DFS and likely OS when added to patients with FLT3-ITD altered AML. The use of midostaurin in FLT3 altered AML in the frontline setting showed improved OS and maintenance midostaurin may also benefit those that responded to induction and consolidation.

Despite the prior shortcomings of maintenance trials in AML, the recent success coupled with improvements in diagnosing, classifying, and treating AML make room for new approaches to maintenance therapy. In the non-alloSCT setting, stratifying patients based on the presence of detectable MRD at the time of consideration of maintenance therapy will help identify patients at high risk for relapse which will allow for better selected patients for trials of maintenance therapy. The continued development of better molecularly and immunologically targeted agents may allow for safer treatment and improved outcomes.

## Author Contributions

Both PR and TK conceived and wrote the manuscript. All authors contributed to the article and approved the submitted version.

## Funding

Dr. Reville is supported by grants from the National Institutes of Health, USA (NIH grants T32CA009666).

## Conflict of Interest

Dr. Kadia reports personal fees from: Novartis, Pfizer, Abbvie, Genentech, JAZZ, Agios and grants from: Pfizer, BMS, Abbvie, Genetech, JAZZ, Amgen, Astra Zeneca, Astellas, Celleknos, Genfleet, DeltaFlyPharma.

The remaining author declares that the research was conducted in the absence of any commercial or financial relationships that could be construed as a potential conflict of interest.
